# Detection of Cytomegalovirus Antibodies Using a Biosensor Based on Imaging Ellipsometry

**DOI:** 10.1371/journal.pone.0136253

**Published:** 2015-08-21

**Authors:** Hongliu Sun, Cai Qi, Yu Niu, Tengfei Kang, Yongxin Wei, Gang Jin, Xianzhi Dong, Chunhua Wang, Wei Zhu

**Affiliations:** 1 School of Pharmaceutical Sciences, Binzhou Medical University, Yantai, China; 2 Institute of Equipment Technology, Chinese Academy of Inspection and Quarantine, #3, Beijing,China; 3 Institute of Mechanics, Chinese Academy of Sciences, #15, Beijing, China; 4 Food Laboratory, Beijing Inspection and Quarantine Testing Center, #6, Beijing, China; 5 Institute of Biophysics, Chinese Academy of Sciences, #15, Beijing, China; 6 Institute of Radiation Medicine, Shandong Academy of Medical Sciences, #18877, Jinan, China; IPK, GERMANY

## Abstract

**Background:**

Cytomegalovirus (CMV) is the most common infectious cause of mental disability in newborns in developed countries. There is an urgent need to establish an early detection and high-throughput screening method for CMV infection using portable detection devices.

**Methods:**

An antibody analysis method is reported for the detection and identification of CMV antibodies in serum using a biosensor based on high spatial resolution imaging ellipsometry (BIE). CMV antigen (CMV-3A) was immobilized on silicon wafers and used to capture CMV antibodies in serum. An antibody against human immunoglobulin G (anti-IgG) was used to confirm the IgG antibody against CMV captured by the CMV-3A.

**Results:**

Our results show that this assay is rapid and specific for the identification of IgG antibody against CMV. Further, patient serum was quantitatively assessed using the standard curve method, and the quantitative results were in agreement with the enzyme-linked immunosorbent assay. The CMV antibody detection sensitivity of BIE reached 0.01 IU/mL.

**Conclusions:**

This novel biosensor may be a valuable diagnostic tool for analysis of IgG antibody against CMV during CMV infection screening.

## Introduction

CMV is the most common infectious cause of mental disability in newborns in developed countries [1]. Detection of CMV antibodies is effective for systematic screening for CMV infection [2]. For instance, the CMV immunoglobulin G (IgG) avidity assay can help to distinguish primary from non-primary human CMV infections [3–5]. The key immune methods used for CMV antibody detection are: enzyme-linked immunosorbent assay (ELISA) [6], Elecsys [7], electrochemiluminescence immunoassay (ECLIA) [8], immunofluorescence assay (IFA) [9], flow cytometry (FCM) [10] and immunoblots [11]. Additionally, new immune methods for CMV antibody detection have been developed, such as the chemiluminesent microparticle immunoassay (CMIA) [12] and protein microarrays [13, 14]. However, these methods suffer from inherent limitations, such as length of testing time, the needs for expensive equipment, specialist skills, low sensitivity, and complicated sample preparation processes. For example, conventional ELISA is still the main diagnostic test for CMV, and commercial CMV ELISA Kits are available. Although ELISA is often used as a comparison method for CMV antibody detection [13], obvious shortcomings include the need of tracer label, plate washing, the indirect format of detection, and the length of time necessary for testing. Thus, a rapid, simple, direct, and high-throughput method for CMV antibody detection is urgently needed.

The first biosensor based on imaging ellipsometry (BIE) was developed in 1995 [15,16]. Compared to the methods above, the advantages of BIE are evident, *e*.*g*., high-throughput multiplexed analysis and quantitative, label-free rapid testing. Previous applications of BIE mainly focus on the biomedical fields [17,18], such as high-throughput disease diagnosis of hepatitis B virus (HBV) marker [19,20], the detection of avian influenza virus (AIV) [21] and antibodies against severe acute respiratory syndrome (SARS) detection [22]. Thus, the biosensor technology offers important tools for disease diagnosis. As such, new applications have recently been developed, including those for the analysis of the interaction between tropomyosin allergens and antibodies [23], and the interaction between soluble N-ethylmaleimide-sensitive factor attachment receptor (SNARE) proteins [24]. To date, however, BIE has not been applied for the detection of CMV antibody, particularly for the identification of the CMV IgG and IgM antibodies.

The purpose of this study was to detect antibodies against CMV-3A in patient serum using a BIE microarray with CMV-3A, as well as to specifically identify captured IgG antibody against CMV. Antibodies against CMV qualitatively were detected using BIE, and then, goat antibody against human IgG (anti-IgG) was added to the area with the captured CMV antibody to confirm IgG antibody against CMV. A standard curve representing different concentration gradients was also established for the quantitative detection of CMV antibodies. As such, the concentration of CMV antibody in serum was quantitatively detected by BIE and then compared using ELISAs.

## Materials and Methods

### Materials and reagents

Silicon wafers were purchased from the General Research Institute for Nonferrous Metals (China). N-hy-droxysuccinimide (NHS) and 1-(3-Dimethyla-minopropyl)-3-ethylcarbodii-mide hydrochloride (EDC), Tween-20, bovine serum albumin (BSA), IgG, and Blocking Buffer (10×, B6429) were purchased by Sigma. Anti-IgG and a goat antibody against human IgM (anti-IgM) were purchased from Beijing Bo Sheng Bio-technology Co. Ltd. CMV-3A was purchased from GalaxyBio. The CMV-3A was a fusion of three segments of Pp150, Gp52 and Pp65, which have strong antigenicity epitopes. Fusion of the multi-epitope both enhances the sensitivity/specificity and reduces false negatives. Thus, CMV-3A was used as the ligand in the BIE assay. Purified CMV antibody (Pp65, C-Term, Rabbit, 1 mg/mL) was purchased from Antibodies-online.com. Patient serum samples were purchased from QiLu Hospital of Shandong University and clinical information is listed in Table A in S2 File.

TORCH ELISA Kits used to analyze serum samples were purchased from MEDSON Inc. The detection process and analysis were executed according to the manufacturer’s instructions. Microplates were coated with native CMV antigens, highly purified by sucrose gradient centrifugation and inactivated. The solid phase was first treated with the diluted sample, and IgG molecules to CMV were then captured, if present, by the antigens. After washing out all of the other sample components, bound anti-CMV IgG molecules were detected by the addition of specific polyclonal anti-H-IgG antibodies labelled with peroxidase (HRP) in the second incubation. The enzyme captured on the solid phase, acting on the substrate/chromogen mixture, generates an optical signal that is proportional to the amount of anti-CMV IgG antibodies present in the sample. A calibration curve, calibrated against the first W.H.O international standard, makes possible a quantitative determination of the IgG antibody in the patient.

NE solution was prepared with NHS (0.05 M) and EDC (0.2 M) in deionized water (18.3 MΩcm) from a Milli-Q plus system (Millipore, Bedford, MA). Phosphate-buffered saline (PBS) was prepared with 140 mM NaCl, 2.7 mM KCl, 10 mM Na_2_HPO_4_, and 1.8 mM KH_2_PO_4_ (pH 7.3) in deionized water. PBST buffer was prepared with 1% Tween-20 in PBS. CMV-3A (0.1 mg/mL) was prepared with PBST. BSA (10 mg/mL) was prepared with PBST. The blocking reagent was a 1:1 (vol:vol) mixture of 10 mg/mL BSA and 10× Blocking Buffer. Purified CMV antibody, IgG, anti-IgG, and anti-IgM were diluted to a concentration of 0.1 mg/mL with PBST. Serum samples were diluted as indicated with PBST for qualitative or quantitative detection.

### BIE principle

The BIE combines high spatial resolution imaging ellipsometry (FigA in S1 File) with a microfluidic system (FigB in S1 File) to analyze biomolecular interaction [25].

The microfluidic system is used for surface patterning and array production, as well as for solution delivery, ligand immobilization and target capture [26]. The microfluidic system has four main parts: a sample plate, multi-cell array, micro-channels and pumps. A multi-cell array was formed when a polydimethyl-siloxane (PDMS) pattern mold contacted the surface of a silicon substrate, each cell having an inlet and an outlet for solution passage. The physical size of each cell was ~1.5×1×0.5 mm^3^. The inlet micro-channels were placed into sample plate, and the outlet micro-channels were connected with pumps (ISM939, ISMATEC, Switzerland. www.ismatec.com) offering negative pressure. The simple channel junctions can be used in serial or parallel formats to simultaneously analyze single or multiple samples.

Imaging ellipsometry is a display technique for ultrathin film and surface characterization [27], which is used to read and analyze the protein arrays made by microfluidic systems. The incident wave of polarized light irradiates the sample as a probe beam and is thereby modified resulting in a reflective or transmission beam having the ability to carry sample information, such as protein layer thickness. When imaging ellipsometry is used to detect layer thickness, the reflection intensity is represented in grayscale, and the variation in layer thickness leads to changes in the grayscale value. If the refractive index is invariant, the grayscale value is directly proportional to the thickness of the protein layer within the range of 0~30 nm layer thickness, *i*.*e*., I = kd, where I is the light intensity and d is the layer thickness [28]. Under these conditions k is a constant and can be determined from a protein layer with known grayscale values and known thickness [29]. There is also a relationship between surface concentration and film thickness: surface concentration (μg/cm^2^)≈K×d, where K = 0.12 [27]. Thus, the grayscale value directly reflects layer thickness and surface concentration. The higher the grayscale value, the thicker the layer and the higher the surface concentration.

### Surface modification

Silicon wafers were cut into 20×10 mm^2^ rectangles and rinsed with deionized water. After soaking in piranha solution (30% H_2_O_2_:98% H_2_SO_4_ = 1:3, vol/vol) for 30 min to increase the number of silanol groups on the wafer surface, and rinsing with deionized water and ethanol, the wafers were soaked in a mixture of 3-aminopropyltriethoxy-silane (APTES) and absolute ethanol (APTES:absolute ethanol = 1:10, vol/vol) and incubated for 2 h with gentle agitation. The reaction of APTES with the surface silanol groups resulted in covalent immobilization of-O-Si(OH)_2_-(CH_2_)_3_-NH_2_, forming a layer of densely packed amino groups on the surface. After rinsing three times with absolute ethanol, the wafers were incubated in a saturated solution of succinic anhydride in ethanol for ≥ 3 h with gentle agitation. The CH_2_CH_2_COOCO- group of succinic anhydride reacted with the -NH_2_ of -O-Si(OH)_2_-(CH_2_)_3_-NH_2_ group immobilized on the surface, generating carboxyl groups (-(CH_2_)_3_NH-CO-(CH_2_)_2_-COOH). Once prepared, the wafers were stored in ethanol.

### CMV detection procedure

The above-modified silicon wafers were used as the substrate. A piece of silicon wafer was placed on the microfluidic mold of the microfluidic system so that surfaces of the wafers were patterned to form small, regular cells in an array format. Then, the carboxyl groups were activated by pumping 10 μL of NE into each cell at a flow rate of 5 μL/min and passed through the surface of the wafer. In the presence of NHS, EDC transfers carboxyl groups to the Sulfo-NHS ester, which reacts with the amino groups of proteins to immobilize the proteins on surface. Next, the ligand was immobilized: CMV-3A as ligand or probe was pumped into each cell (10 μL per cell at 1 μL/min). Third, blocking occurred by pumping the blocking reagent into each cell (50 μL per cell at 1 μL/min) and passing it through each ligand area. Thus, a sensing array surface with CMV-3A was formed to catch CMV antibodies. Fourth, targets were detected. PBST was used as blank control (50 μL per cell at 1 μL/min) and purified CMV antibody as a positive control (50 μL per cell at 1 μL/min) by pumping them into several of the individual cells. Simultaneously, patient serum samples were also pumped into remaining cells (50 μL per cell at 1 μL/min). The CMV antibodies in the serum were captured when they interacted with the CMV-3A on the sensing surface. All cells were rinsed with PBST (20 μL per cell at 20 μL/min) between every two consecutive operation steps. Finally, the microarray wafers were removed from the microfluidic system. After being rinsed with deionized water and dried with nitrogen, the wafers were analyzed using imaging ellipsometry. If the CMV antibodies in the solution or serum interacted with the CMV-3A on the surface and formed a complex, the layer in that area became thicker. The experimental results were recorded as images in grayscale, and binding of CMV antibodies resulted in a significant increase in grayscale value.

### Qualitative detection of CMV antibodies and patient serum

**Fig 1:**
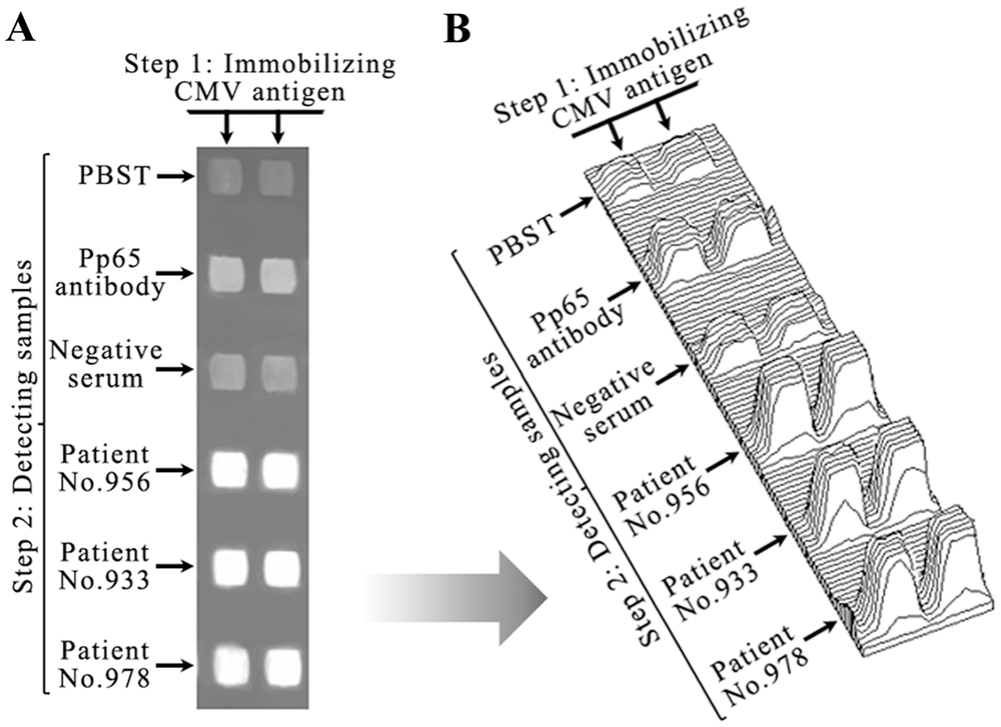
Specificity and qualitative detection of CMV antibodies using BIE. (A) Grayscale images of different CMV antibodies samples; and (B) 3-D grayscale distribution map of different CMV antibody samples in image A. In the first step, CMV-3A was immobilized as the ligand on two columns. In the second step, PBST buffer was added as a blank control to two areas on the first row. Simultaneously, purified CMV antibody was added as a positive control to two areas on the second row. Normal serum without CMV antibodies was added as a negative control to two areas on the third row. According to ELISA data, No. 956 had a high CMV antibody concentration, and No. 933 and 978 had medium CMV antibody concentrations. To observe qualitative detection of CMV antibodies in serum, samples with higher concentrations were chosen.

To detect the specificity of antibodies against CMV, CMV-3A as ligand was immobilized on two columns (Fig 1). PBST buffer was added as blank control to two areas on the first row. Simultaneously, purified CMV antibody (0.1 mg/mL) was added as positive control to two areas on the second row. Normal serum without CMV antibodies was added as negative control to two areas on the third row. Three patient serum samples (No. 956, 933, and 978; see Table A in S2 File for sample information) were analyzed on the following rows, respectively. The increase in light reflection density at each area revealed that CMV antibodies in the samples interacted with the CMV-3A immobilized on the chip.

**Fig 2:**
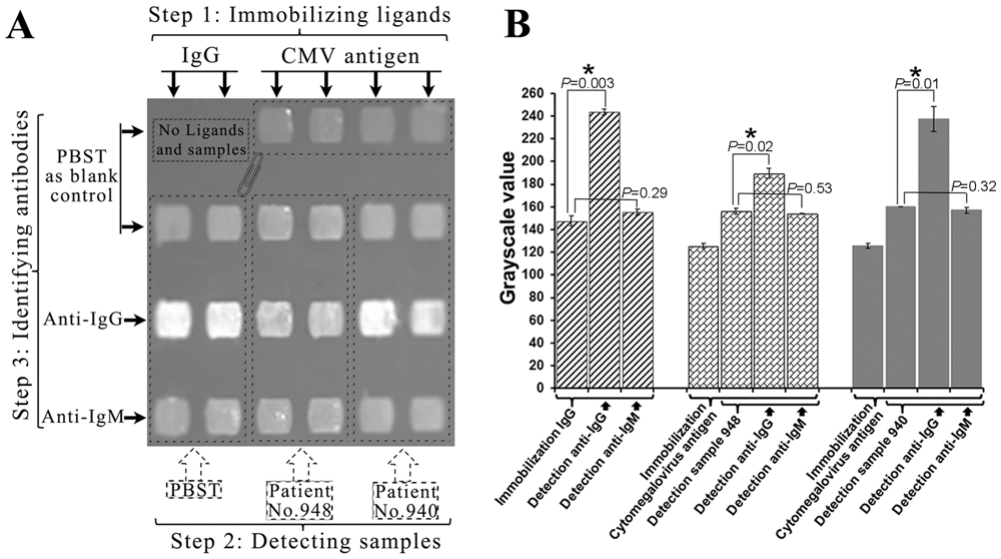
Identification of IgG antibody against CMV by BIE. (A) Grayscale images of IgG antibody identification in serum; and (B) grayscale value and *P*-value of the IgG antibody identification. “*” indicates significant changes in grayscale values. In the first step, IgG was immobilized as ligand on the first and second columns. CMV-3A was immobilized on the third, fourth, fifth, and sixth columns. In the second step, PBST buffer was added as blank control to the corresponding areas in the image. Sample No. 948 was added to the third and fourth columns, and sample No. 940 was added to the fifth and sixth columns. In the third step, PBST buffer was added as blank control to the first areas in every column. Anti-IgG and anti-IgM were added to the third and fourth rows, respectively.

We further analyzed the types of CMV antibodies captured by the CMV-3A (Fig 2). In the first step, IgG was immobilized as ligand on the first and second columns. Simultaneously, CMV-3A was immobilized on the third, fourth, fifth, and sixth columns. In the second step, sample No. 948 was added to the third and fourth columns, and sample No. 940 was added to the fifth and sixth columns. PBST buffer was added as blank control to remaining areas. In the third step, anti-IgG and anti-IgM were added to third and fourth rows to identify and confirm if the antibody captured by the ligand was IgG or IgM. PBST buffer was added as blank control to remaining areas.

### Calibration curve and quantitative detection of clinical serum samples

**Fig 3:**
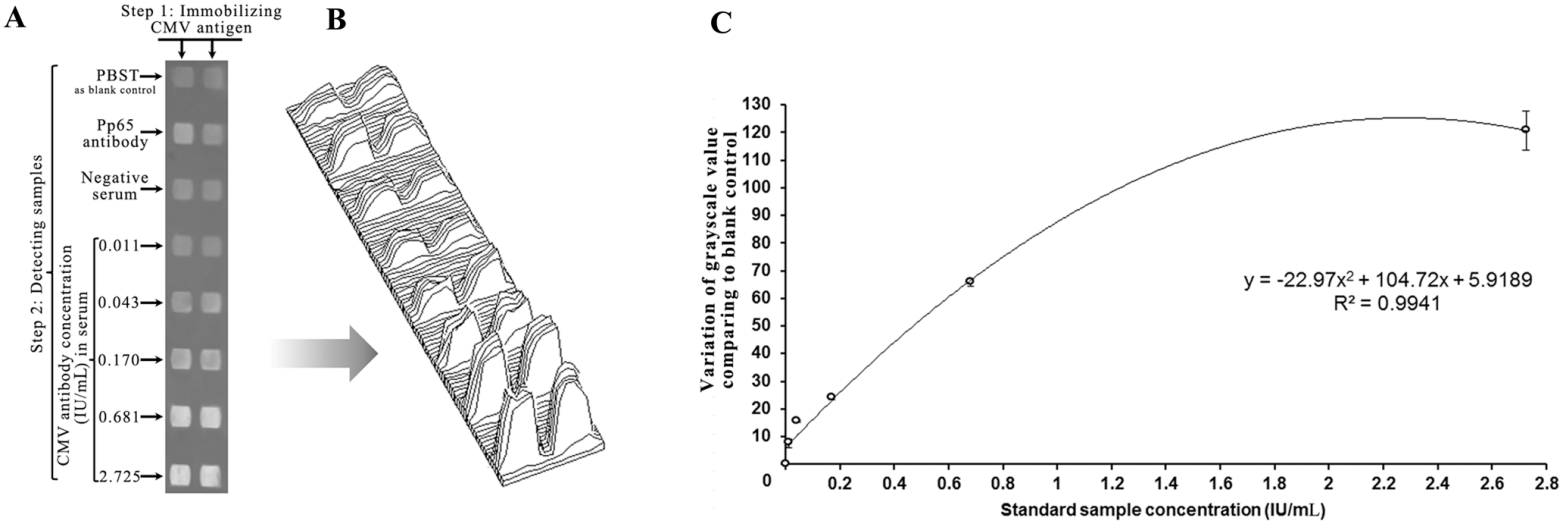
Calibration curve and sensitivity of BIE for detecting CMV antibodies. (A) Grayscale images of different CMV antibody concentration levels detected; (B) 3-D grayscale distribution map of different concentrations of CMV antibodies detected; and (C) standard curve of CMV-3A as ligand to detect five serial dilutions of containing CMV antibodies (0.011, 0.043, 0.170, 0.681, and 2.725 IU/mL). In the first step, CMV-3A was immobilized as the ligand on two columns. In the second step, PBST buffer was added as a blank control to two areas on the first row. Simultaneously, purified CMV antibody was added as a positive control to two areas on the second row. Negative serum was added as a negative control to two areas on the third row. The serial dilutions of CMV antibodies were added as analytical samples on the following rows. The same concentration was measured in two duplicate areas.

To establish a calibration curve, a serum sample (No. 942, 21.8 IU/ml, see Table A in S2 File) was used, and five levels of serial dilution containing 0.011, 0.043, 0.170, 0.681, and 2.725 IU/mL of CMV antibody were prepared in PBST. CMV-3A was immobilized as ligand on two columns (Fig 3). After blocking, PBST buffer, CMV antibody and normal serum without CMV antibody were added as blank, positive, and negative controls (respectively) to the first, second, and third rows, respectively. The 0.011-, 0.043-, 0.170-, 0.681- and 2.725-IU/mL samples were added from the fourth to the eighth rows, respectively. Concentration plotted on the X-axis and the variation of the grayscale value compared to blank control was plotted on the Y-axis to generate the calibration curve (Fig 3).

**Fig 4:**
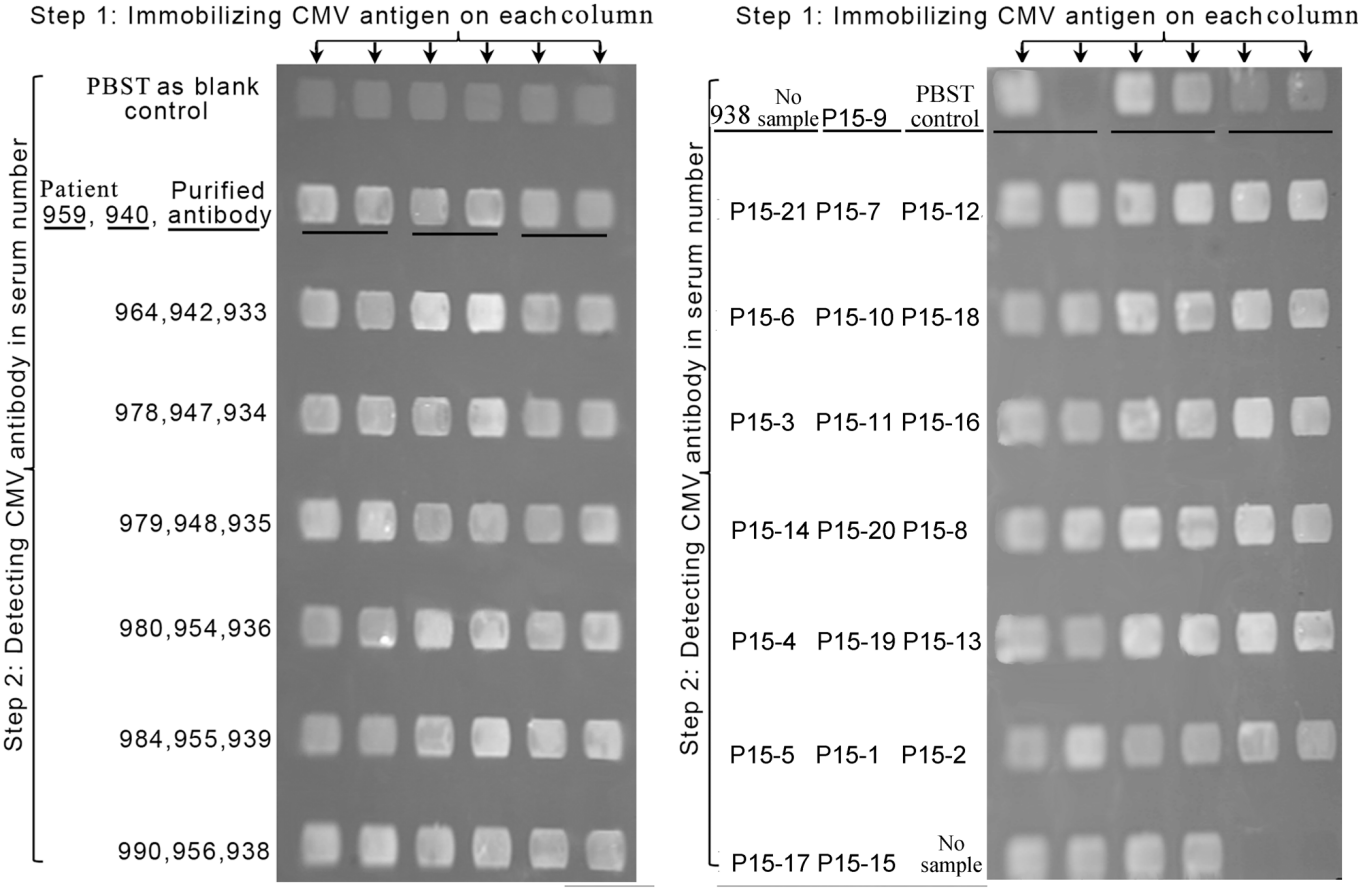
Detection of CMV antibodies in patient serum using BIE. In the first step, CMV-3A was immobilized as the ligand on six columns. In the second step, PBST buffer was added as a blank control to six areas on the first row. Simultaneously, purified CMV antibody was added as a positive control to the last two areas on the second row. Patient serum samples were added as analytical samples on the following areas, respectively. The same serum sample was measured in two duplicate areas (No.940, 959,938 no sample, P15-9, and PBST control are underlined).

After acquiring the calibration curve, the concentrations of antibodies in samples of unknown concentration could be determined on the curve according to their grayscale values. Measurements of every sample were repeated in two areas on one chip (Fig 4). Commercial ELISA CMV antibody kits were used as controls according to the manufacturer’s instructions.

### Statistical analyses

For statistical analyses, the corresponding *P*-values were calculated with single factor analysis of variance in Microsoft Office Excel according to the quantity of areas in images and patients’ serum samples in Table A in S2 File [30]. In qualitative and quantitative detection experiments, significant changes in grayscale value detection areas compared to the blank control areas and the detection areas were deemed positive signals if *P*-value was < 0.05. If the *P* was ≥ 0.05, the detection areas were deemed negative signals. In comparison of the ELISA and BIE data, the results of the two methods were deemed in agreement if *P* was ≥ 0.05. If *P* was < 0.05, the two methods were deemed in disagreement. The correlation coefficient (*r*-value) of BIE and ELISA was calculated with analysis of correlation coefficient in Microsoft Office Excel (Table B in S2 File).

## Results

### Specificity confirmation and qualitative detection

Compared to blank controls areas, the purified CMV antibody and patient serum sample detection areas had markedly thicker films, with the average grayscale value displaying significant increases, while negative control areas did not (Fig 1). The mean grayscale value of blank controls was measured at 117.45 ± 0.92, and the value of the purified CMV antibody was measured at 176.8 ± 3.39. Thus, the mean grayscale value of purified CMV antibody minus the mean grayscale value of blank control was 59.4 (*P =* 0.002). The value of the negative control was 125.9 ± 3.18. The mean grayscale value of the negative control minus the mean grayscale value of the blank control was 8.4 (*P =* 0.07). This indicated that there were specific interactions between the purified CMV antibody and the CMV-3A. Therefore, CMV antibodies in patient samples could be captured by the CMV-3A immobilized on the substrate.

Indeed, the average grayscale value of the serum samples analyzed significantly increased relative to the controls (Fig 1). The mean serum value was 253.58 ± 0.49, and the mean grayscale value of serum minus the mean grayscale value of the blank control was 136.1 (*P* = 2.8×10^−5^), indicating that CMV antibodies were abundant in the serum samples. This was consistent with the ELISA results (Table A in S2 File).

### Identification of IgG antibody against CMV by BIE

When purified human IgG was used as the ligand, the average grayscale value of the anti-IgG detection areas significantly increased, while the anti-IgM detection areas did not (left two columns in Fig 2). The mean grayscale value of the anti-IgG minus the mean grayscale value of the blank control was 96.2 (*P* = 0.003). The mean value of the anti-IgM minus the mean grayscale value of the blank control was 8.15 (*P* = 0.29). These data are indicative of the specific interactions between the anti-IgG and the human IgG immobilized on the chip, which could be used as references to determine whether the antibodies in samples are IgG.

Compared to the CMV antibody areas, the anti-IgG detection areas displayed significant increases, while the anti-IgM detection areas did not (Fig 2). For example, the value of sample 948 was 156.2 ± 2.6, while the value of the anti-IgG was 189.6 ± 4.4, for a difference of 33.4 (*P* = 0.02). However, the value of the anti-IgM was 154.25 ± 0.25, and the difference was not obvious (*P* = 0.53). This indicated that IgG CMV antibodies were captured by the chip. For sample No. 940 detection, the value of the anti-IgG was 237.7 ± 11, and the increase was 77.3 (*P* = 0.01). The result also indicated that the content of IgG in samples No. 948 and 940 was different.

### BIE sensitivity and calibration curve

Five concentration gradients of a serum sample (No. 942, 21.8 IU/mL, see Table A in S2 File) measured in serially diluted samples were used to determine the sensitivity of the BIE assay (Fig 3). We found that the change in signal intensity was consistent with the increase in CMV antibody concentration. The CMV antibody detection sensitivity levels reached 0.01 IU/mL. The grayscale values of the blank control were 124.7 ± 0.5, and the grayscale values of the dilutions as low as 0.01 IU/mL were 132.3 ± 0.1, *i*.*e*., ~ 6.1% greater than that of the controls (*P* = 0.03). Repeating these tests > 10 times, our results demonstrated that the sensitivity of the assay reached 0.01 IU/mL or less. Each variation in grayscale value was linked to a corresponding concentration of the CMV antibody over the range of 0.011–2.725 IU/mL, and the calibration curve is shown in Fig 3. The concentrations of unknown samples could be determined according to corresponding grayscale value on the calibration curve.

### Quantitative detection of CMV antibodies in clinical serum

**Fig 5:**
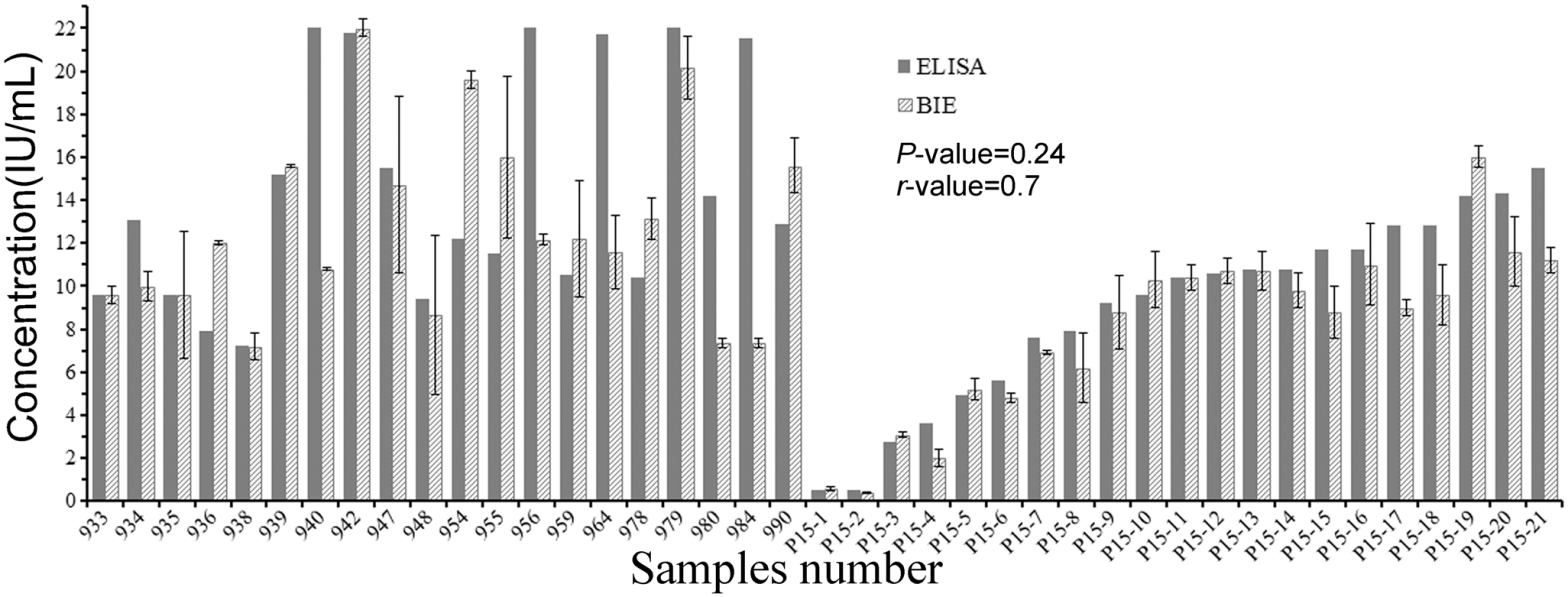
Comparison of BIE with ELISA. P15-1 and P15-2 were used as healthy controls. Concentration of CMV antibody (0.5 IU/mL) located in the normal reference range (0.4–0.6 IU/mL). The correlation coefficient (*r*-value) and *P*-value were calculated.

41 CMV patients (Table A in S2 File) with quantitative results by ELISA were tested with BIE (Fig 4). For different serum samples, the changes in BIE signal intensity were different. The comparison of results between the two methods is shown in Fig 5. Single factor analysis and correlation coefficient analysis revealed that the results were in agreement between ELISA and BIE (F = 1.38<F_0.05_ = 3.96; *P* = 0.24>0.05, r = 0.7) (Table B in S2 File).

## Discussion

High-throughput detection is suitable for mass CMV screening in women of childbearing age. When coupled with microfluidic technologies, BIE can greatly improve the throughput for CMV detection on one chip. The microarrays developed here have multiple cells immobilized with different ligands for different CMV antibody subtypes. Imaging ellipsometry is also suitable to high-throughput analysis. It can be used to visualize the variation in signal from all units of the microarray with high spatial resolution. Patient sample analysis results from one microarray can simultaneously be identified, and the data can be obtained in several seconds using an imaging ellipsometer. Presently, our method can provide simultaneous 48 reaction areas. With its enhanced throughput and lower cost, BIE may be used in clinical mass CMV screenings for healthy births in the future.

Antibody detection is widely available for the clinical diagnosis of CMV infection. However, the identification of antibody types may help to determine the course of disease, and BIE may be used as a clinical primary screening tool for CMV patients. IgM is produced in large amounts early in infection (reaching a peak in the first month), and is followed by IgG production [7,31]. Levels of IgM decline in the months following the onset of infection, whereas IgG levels persist for the rest of the patient’s life [32,33]. Determining IgG avidity can provide additional guidance on infection status, and low avidity IgG is initially present but increases over time [34]. A positive result for IgM combined with low-to-moderate IgG suggests avidity a primary CMV infection within the past 3 to 4 months [35]. The visualized BIE image (Fig 2) could reflect types via difference in brightness, so this technology can achieve rapid screening. To date, our work is simply a demonstration for IgG type antibody detection with BIE. IgM and other antibodies needed to screen for specific antigen can be incorporated in the future for further practical applications.

Presently, most methods mention the relative sensitivity (%) of detection for CMV antibodies [8,36], but little in provided about absolute sensitivity. Compared other methods, the absolute sensitivity of BIE can reach 0.01 IU/mL, and it has good resolution in the range of 0.1–1.0 IU/mL on the calibration curve for quantitative detection. The reference range for detection of CMV antibodies has previously been published. For example, Elecsys (Roche Diagnostics) immunoassays have an equivocal range (0.5–1.0 IU/mL) of CMV IgG [12]. In our experiment, ELISA as a control method had a reference range of 0.4–0.6 IU/mL. Therefore, the sensitivity of our BIE biosensor is below the reference range and has already reached clinical standards.

However, there is a substantial discordance between the ELISA and the BIE in some patients (i.e., 940, 956, 964, 984, and 980). Samples from these patients displayed stronger signal using ELISA than BIE. In ELISAs, anti-CMV IgG molecules are detected by the addition of polyclonal specific anti-H-IgG antibodies labelled with horseradish peroxidase (HRP). In contrast, the BIE biosensor directly identified the variation of the surface anti-CMV IgG concentration after capturing target without a secondary label. Thus, this label-free method may avoid some interference factor. In addition, the grayscale images offered uniform areas, helping to avoid false positive signals. When the correlation coefficient (*r*-value) and *P*-value were calculated after excluding the number of severe mutations (940, 956, 964, and 984), *r*-value = 0.93 and *P* = 0.8 the statistical results showed more agreement between ELISA and BIE.

In conclusion, we have developed a label-free and multiplex screening CMV IgG biosensor method. The high-throughput detection is suitable for mass CMV screening of women of childbearing age. Further, our results demonstrate that the sensitivity of BIE has already reached clinical diagnose standards for anti-CMV IgG. Thus, on the basis of anti-CMV IgG detection, BIE can be used for qualitative and quantitative detection of more types of CMV antibodies using a simple and fast procedure.

## Supporting Information

S1 File **Contains Fig A. Imaging ellipsometry.** (a) Imaging principle [23]; and (b) Laboratory prototype. **Fig B. Microfluidic system.** (a) Internal structure; and (b) Laboratory prototype.
(DOCX)

S2 File Contains Table A. Clinical information of CMV patients from QiLu Hospital of Shandong University. Table B. Statistical analysis process of comparison between the ELISA and BIE data.
(DOCX)
