# Supplementary material for: Detection of Cytomegalovirus Antibodies Using a Biosensor Based on Imaging Ellipsometry
Source: PLoS One. 2015 Aug 21;10(8):e0136253. doi: 10.1371/journal.pone.0136253 (PMC4546680; doi:10.1371/journal.pone.0136253)
Supplement: S1 File — Contains Fig A. Imaging ellipsometry. (a) Imaging principle [23]; and (b) Laboratory prototype. Fig B. Microfluidic system. (a) Internal structure; and (b) Laboratory prototype. (DOCX) [file pone.0136253.s001.docx]

Running title: Cytomegalovirus detection using biosensors.

# Detection of cytomegalovirus antibodies using a biosensor based on imaging ellipsometry

Hongliu Sun^1,#^, Cai Qi ^2,#^, Yu Niu^3^, Tengfei Kang^3^, Yongxin Wei^4^, Gang Jin^3^, Xianzhi Dong^5^, Chunhua Wang^1^, Wei Zhu^6,^*

*^1^ School of Pharmaceutical Sciences, Binzhou Medical University, #346, Guanhai Rd., Yantai, 264003, China*

*^2^ Institute of Equipment Technology, Chinese Academy of Inspection and Quarantine, #3, Gaobeidian North Rd., Beijing, 100123,China*

*^3^ Institute of Mechanics, Chinese Academy of Sciences, #15, Beisihuan West Rd., Beijing, 100190, China*

*^4^ Food Laboratory, Beijing Inspection and Quarantine Testing Center. #6, Tianshuiyuan Rd., Beijing, 100026, China*

*^5^ Institute of Biophysics, Chinese Academy of Sciences, #15, Datun Rd., Beijing, 100101, China*

*^6^ Institute of Radiation Medicine, Shandong Academy of Medical Sciences, #18877, Jingshi Rd., Jinan, 250062, China*

* Corresponding author. Tel/fax: 86-531-82919956. *E-mail address:* [fsszw@163.com](mailto:fsszw@163.com)

# These authors contributed equally to this work.


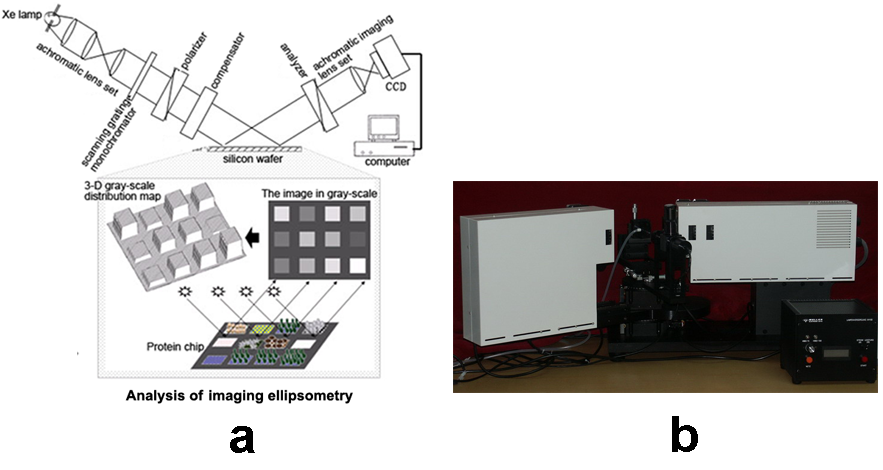


Fig.A. Imaging ellipsometry. (a) Imaging principle [Wang W,et al.Anal.Chem.2013,85:4446.]; and (b) Laboratory prototype.


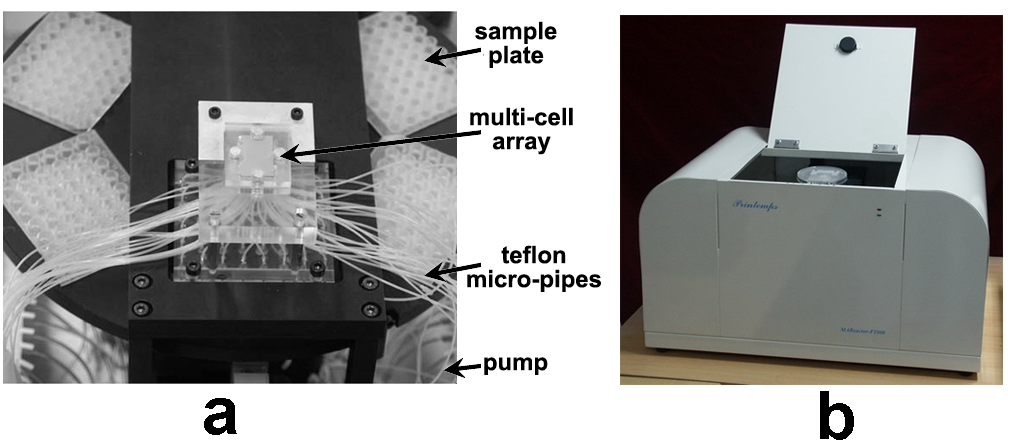


Fig. B. Microfluidic system. (a) Internal structure; and (b) Laboratory prototype.
