# Supplementary material for: Detection of Cytomegalovirus Antibodies Using a Biosensor Based on Imaging Ellipsometry
Source: PLoS One. 2015 Aug 21;10(8):e0136253. doi: 10.1371/journal.pone.0136253 (PMC4546680; doi:10.1371/journal.pone.0136253)
Supplement: S2 File — Contains Table A. Clinical information of CMV patients from QiLu Hospital of Shandong University. Table B. Statistical analysis process of comparison between the ELISA and BIE data. (DOCX) [file pone.0136253.s002.docx]

Running title: Cytomegalovirus detection using biosensors.

# Detection of cytomegalovirus antibodies using a biosensor based on imaging ellipsometry

Hongliu Sun^1,#^, Cai Qi ^2,#^, Yu Niu^3^, Tengfei Kang^3^, Yongxin Wei^4^, Gang Jin^3^, Xianzhi Dong^5^, Chunhua Wang^1^, Wei Zhu^6,^*

*^1^ School of Pharmaceutical Sciences, Binzhou Medical University, #346, Guanhai Rd., Yantai, 264003, China*

*^2^ Institute of Equipment Technology, Chinese Academy of Inspection and Quarantine, #3, Gaobeidian North Rd., Beijing, 100123,China*

*^3^ Institute of Mechanics, Chinese Academy of Sciences, #15, Beisihuan West Rd., Beijing, 100190, China*

*^4^ Food Laboratory, Beijing Inspection and Quarantine Testing Center. #6, Tianshuiyuan Rd., Beijing, 100026, China*

*^5^ Institute of Biophysics, Chinese Academy of Sciences, #15, Datun Rd., Beijing, 100101, China*

*^6^ Institute of Radiation Medicine, Shandong Academy of Medical Sciences, #18877, Jingshi Rd., Jinan, 250062, China*

* Corresponding author. Tel/fax: 86-531-82919956. *E-mail address:* [fsszw@163.com](mailto:fsszw@163.com)

# These authors contributed equally to this work.**Table A. Clinical information of CMV patients from QiLu Hospital of Shandong University.**

| **Patient number^a^** | **Age** | **F: Female; M: Male** | **ELISA detection concentration of CMV IgG (IU/mL)** |
| --- | --- | --- | --- |
| 933 | 29 | F | 9.6 |
| 934 | 55 | M | 13.1 |
| 935 | 25 | F | 9.6 |
| 936 | 31 | F | 7.9 |
| 938 | 43 | F | 7.2 |
| 939 | 30 | F | 15.2 |
| 940 | 25 | F | 22 |
| 942 | 27 | F | 21.8 |
| 947 | 25 | F | 15.5 |
| 948 | 28 | F | 9.4 |
| 954 | 25 | F | 12.2 |
| 955 | 30 | F | 11.5 |
| 956 | 30 | F | >22 |
| 959 | 27 | F | 10.5 |
| 964 | 59 | F | 21.7 |
| 978 | 23 | F | 10.4 |
| 979 | 29 | F | >22 |
| 980 | 30 | F | 14.2 |
| 984 | 27 | F | 21.5 |
| 990 | 26 | F | 12.9 |
| P15-1 | 28 | F | 0.5 |
| P15-2 | 31 | F | 0.5 |
| P15-3 | 27 | F | 2.8 |
| P15-4 | 29 | F | 3.6 |
| P15-5 | 31 | F | 4.9 |
| P15-6 | 25 | F | 5.6 |
| P15-7 | 27 | F | 7.6 |
| P15-8 | 25 | F | 7.9 |
| P15-9 | 27 | F | 9.2 |
| P15-10 | 32 | F | 9.6 |
| P15-11 | 32 | F | 10.4 |
| P15-12 | 31 | F | 10.6 |
| P15-13 | 25 | F | 10.8 |
| P15-14 | 27 | F | 10.8 |
| P15-15 | 27 | F | 11.5 |
| P15-16 | 31 | F | 11.7 |
| P15-17 | 29 | F | 12.8 |
| P15-18 | 25 | F | 13.3 |
| P15-19 | 25 | F | 14.2 |
| P15-20 | 27 | F | 14.3 |
| P15-21 | 31 | F | 15.5 |

^a^ 41 patients were detected with ELISA Kits purchased by MEDSON Inc (<http://www.medsoninc.com>). Normal reference range is 0.4-0.6 IU/mL.

**Table B. Statistical analysis process of comparison between the ELISA and BIE data**

| Difference source | SS | df | MS | F | P-value | F crit |
| --- | --- | --- | --- | --- | --- | --- |
| Difference between groups | 37.6452878 | 1 | 37.64529 | 1.38136 | 0.24336 | 3.96035 |
| Difference inter-group | 2180.194693 | 80 | 27.25243 |  |  |  |
| Total | 2217.83998 | 81 |  |  |  |  |
